# Training curriculum psychiatry: an European perspective

**DOI:** 10.1192/j.eurpsy.2024.39

**Published:** 2024-08-27

**Authors:** A. Szulc

**Affiliations:** Department of Psychiatry, Medical University of Warsaw, Warsaw, Poland

## Abstract

**Abstract:**

Training in psychiatry varies greatly from country to country in Europe - there are differences in the duration of training, the content of training, etc. Different perspectives on training will be presented, especially as far as common features are concerned. We will also present proposals and directions leading to a common European curriculum in psychiatry.Further work is needed in the direction of developing a European curriculum and organizing a European exam in psychiatry.

**Disclosure of Interest:**

None Declared

